# Supplementary material for: Nurses’ occupational fatigue level and risk factors: A systematic review and meta-analysis
Source: PLoS One. 2025 Jul 18;20(7):e0326519. doi: 10.1371/journal.pone.0326519 (PMC12273991; doi:10.1371/journal.pone.0326519)
Supplement: S1 Table — (DOCX) [file pone.0326519.s001.docx]

**Table S1** Systematic literature review search strategy in PubMed

| **Database** | **Index and keyword terms** |
| --- | --- |
| PubMed | #1TS = (Nurses OR Nurse OR Personnel, Nursing OR Nursing Personnel OR Registered Nurses OR Nurse, Registered OR Nurses, Registered OR Registered Nurse)  #2TS = (work fatigue) OR (occupational fatigue) OR (workplace fatigue) OR (tiredness) OR (chronic fatigue) OR (acute fatigue)  #3TS = #3(risk factor*) OR (hazard factor*) OR (dangerous factor*)  #1 AND #2 AND #3 |
