# Supplementary material for: Nurses’ occupational fatigue level and risk factors: A systematic review and meta-analysis
Source: PLoS One. 2025 Jul 18;20(7):e0326519. doi: 10.1371/journal.pone.0326519 (PMC12273991; doi:10.1371/journal.pone.0326519)
Supplement: S2 Table — (DOCX) [file pone.0326519.s002.docx]

**Table S2** Quality assessment for each study

| Study | Item 1 | Item 2 | Item 3 | Item 4 | Item 5 | Item 6 | Item 7 | Item 8 | Item 9 | Item 10 | Item 11 | Total scores | Quality |
| --- | --- | --- | --- | --- | --- | --- | --- | --- | --- | --- | --- | --- | --- |
| Chen (2023) | 1 | 1 | 1 | 1 | 1 | N | 1 | 1 | N | 1 | U | 8 | High |
| Yamaguchi (2023) | 1 | 1 | 1 | 1 | 1 | N | 1 | 1 | 1 | 1 | U | 9 | High |
| Cho (2022) | 1 | 1 | 1 | 1 | 1 | N | 1 | 1 | 1 | 1 | U | 9 | High |
| Min (2022) | 1 | 1 | 1 | 1 | 1 | N | 1 | 1 | N | N | U | 7 | Middle |
| Cho (2022) | 1 | 1 | 1 | 1 | 1 | N | 1 | 1 | 1 | 1 | U | 9 | High |
| Sagherian (2022) | 1 | 1 | 1 | 1 | 1 | N | 1 | N | 1 | 1 | U | 8 | High |
| Alsayed (2022) | 1 | 1 | 1 | 1 | 1 | N | 1 | N | 1 | 1 | U | 8 | High |
| Qian (2022) | 1 | 1 | 1 | 1 | 1 | N | 1 | 1 | N | 1 | U | 8 | High |
| Alshammari (2022) | 1 | 1 | 1 | 1 | 1 | N | 1 | N | N | N | U | 6 | Middle |
| Bolkan Günaydın (2022) | 1 | N | 1 | 1 | 1 | N | 1 | N | N | N | U | 5 | Middle |
| Ross (2021) | 1 | N | 1 | 1 | 1 | N | N | 1 | N | 1 | U | 6 | Middle |
| Hong (2021) | 1 | N | 1 | 1 | 1 | N | 1 | N | 1 | 1 | U | 7 | Middle |
| Min (2021) | 1 | N | 1 | 1 | 1 | N | 1 | 1 | 1 | 1 | U | 8 | High |
| Mollica (2021) | 1 | N | 1 | 1 | 1 | N | 1 | 1 | 1 | 1 | U | 8 | High |
| Orique (2019) | 1 | 1 | 1 | 1 | 1 | N | N | 1 | 1 | 1 | U | 8 | High |
| Ismail (2019) | 1 | N | 1 | 1 | 1 | N | 1 | 1 | N | 1 | U | 7 | Middle |
| Yu (2019) | 1 | N | 1 | 1 | 1 | N | 1 | N | 1 | 1 | U | 7 | Middle |
| Min (2019) | 1 | 1 | 1 | 1 | 1 | N | 1 | N | 1 | 1 | U | 8 | High |
| Sagherian (2017) | 1 | N | 1 | 1 | 1 | N | 1 | N | 1 | 1 | U | 7 | Middle |
| Blouin (2016) | 1 | N | 1 | 1 | 1 | N | N | N | N | 1 | U | 5 | Middle |
| Drake (2016) | 1 | N | 1 | 1 | 1 | N | 1 | N | N | 1 | U | 6 | Middle |
| Liu (2016) | 1 | N | 1 | 1 | 1 | N | 1 | N | 1 | 1 | U | 7 | Middle |
| Zhou (2015) | 1 | 1 | 1 | 1 | 1 | N | 1 | 1 | N | 1 | U | 8 | High |
| Chen (2014) | 1 | 1 | 1 | 1 | 1 | N | 1 | 1 | N | 1 | U | 8 | High |
| Hazzard (2013) | 1 | N | 1 | 1 | 1 | N | 1 | N | N | 1 | U | 6 | Middle |
| Geiger-Brown (2012) | 1 | N | 1 | 1 | 1 | N | 1 | 1 | 1 | 1 | U | 8 | High |
| Barker (2011) | 1 | N | 1 | 1 | 1 | N | N | 1 | N | 1 | U | 6 | Middle |
| Fang (2009) | 1 | 1 | 1 | 1 | 1 | N | N | 1 | N | 1 | U | 7 | Middle |

*Note: Y, yes; N, no; U, unclear.*

Item 1: Define the source of information (survey, record review).

Item 2: List inclusion and exclusion criteria for exposed and unexposed subjects (cases and controls) or refer to previous publications.

Item 3: Indicate time period used for identifying patients.

Item 4: Indicate whether or not subjects were consecutive if not population-based.

Item 5: Indicate if evaluators of subjective components of study were masked to other aspects of the status of the participants.

Item 6: Describe any assessments undertaken for quality assurance purposes (e.g., test/retest of primary outcome measurements).

Item 7: Explain any patient exclusions from analysis.

Item 8: Describe how confounding was assessed and/or controlled.

Item 9: If applicable, explain how missing data were handled in the analysis.

Item 10: Summarize patient response rates and completeness of data collection.

Item 11: Clarify what follow-up, if any, was expected and the percentage of patients for which incomplete data or follow-up was obtained.
