# Supplementary figures and images for: Nurses’ occupational fatigue level and risk factors: A systematic review and meta-analysis
Source: PLoS One. 2025 Jul 18;20(7):e0326519. doi: 10.1371/journal.pone.0326519 (PMC12273991; doi:10.1371/journal.pone.0326519)

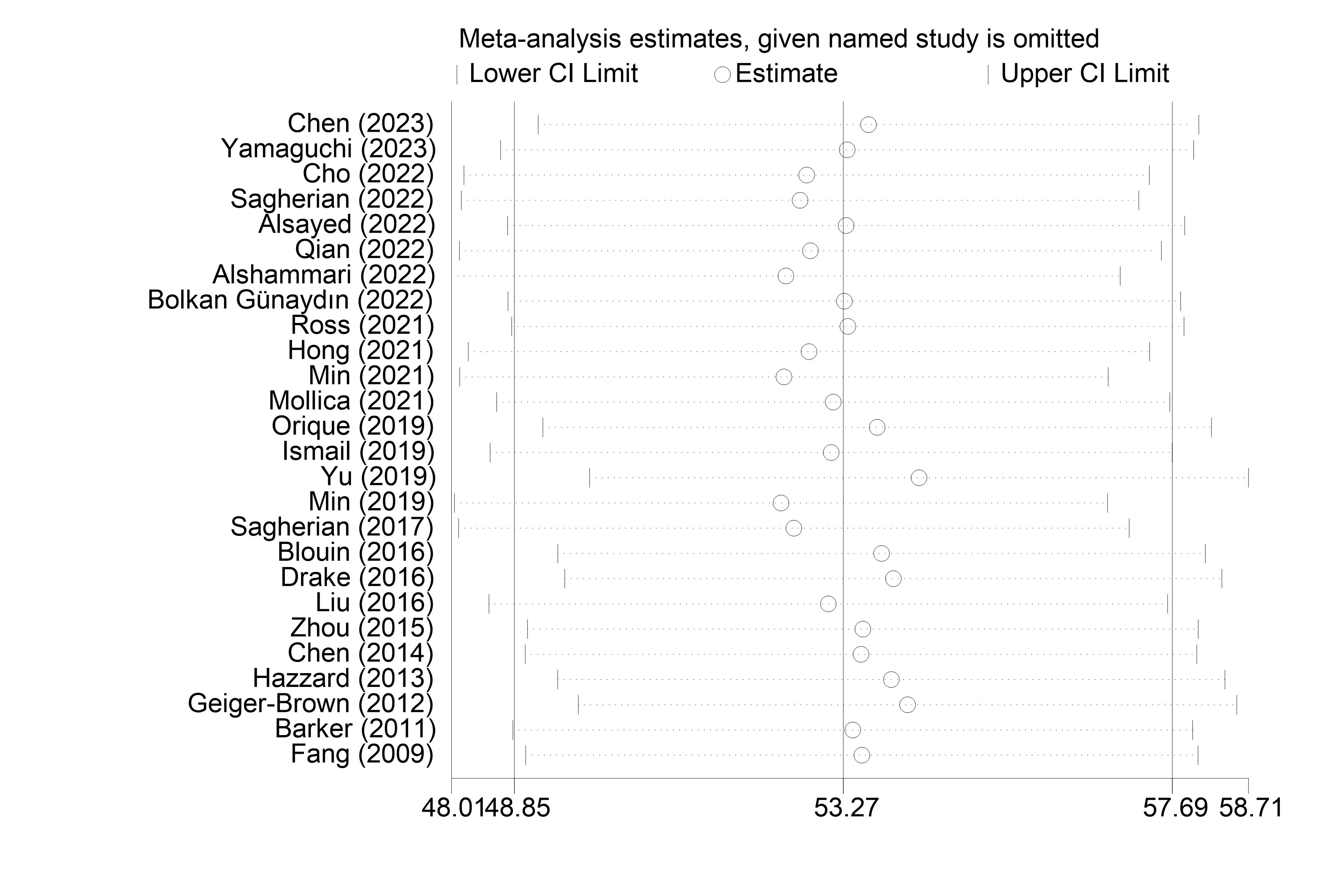

Supplement: S1 Fig — (TIFF) [file pone.0326519.s004.tiff]

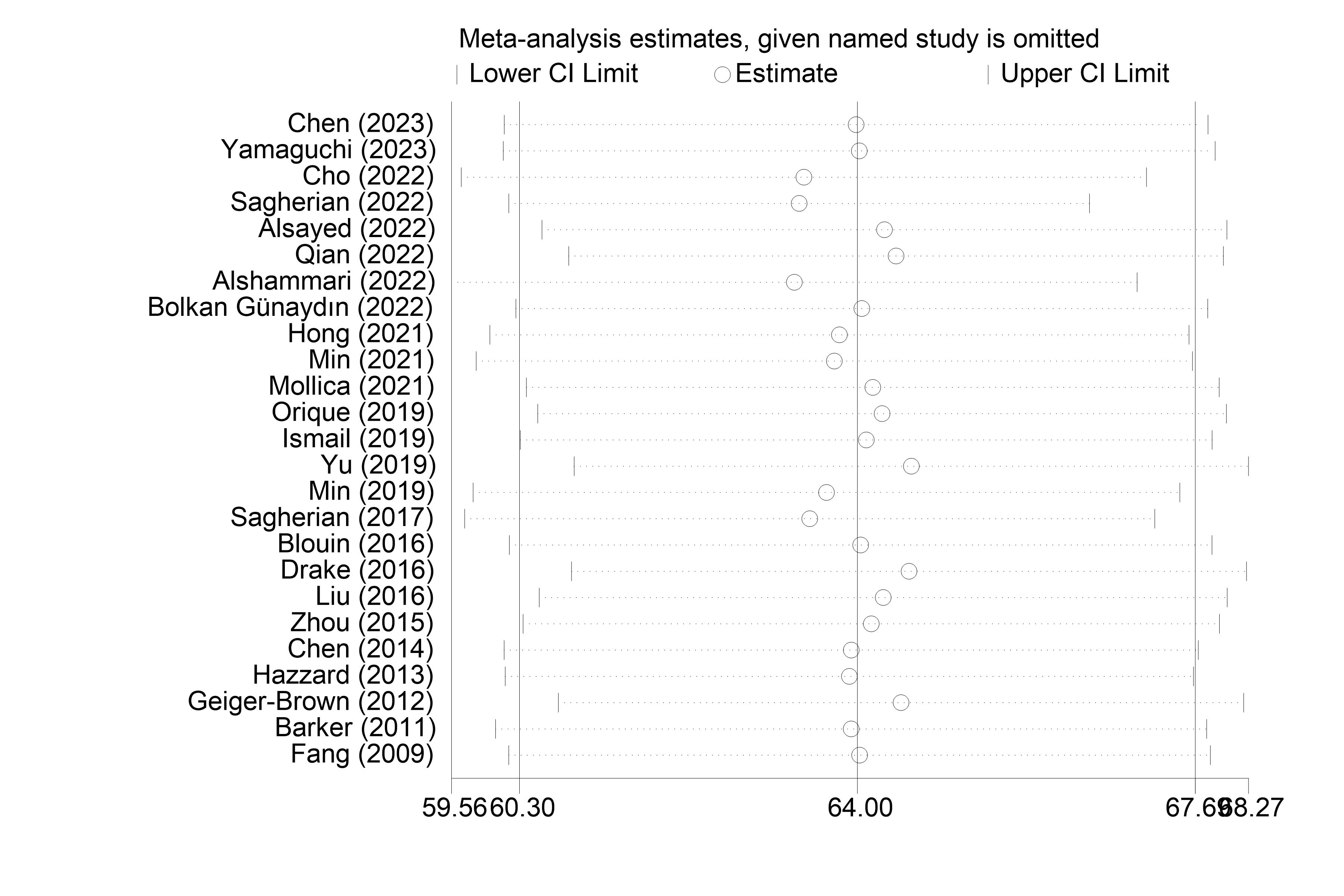

Supplement: S2 Fig — (TIFF) [file pone.0326519.s005.tiff]

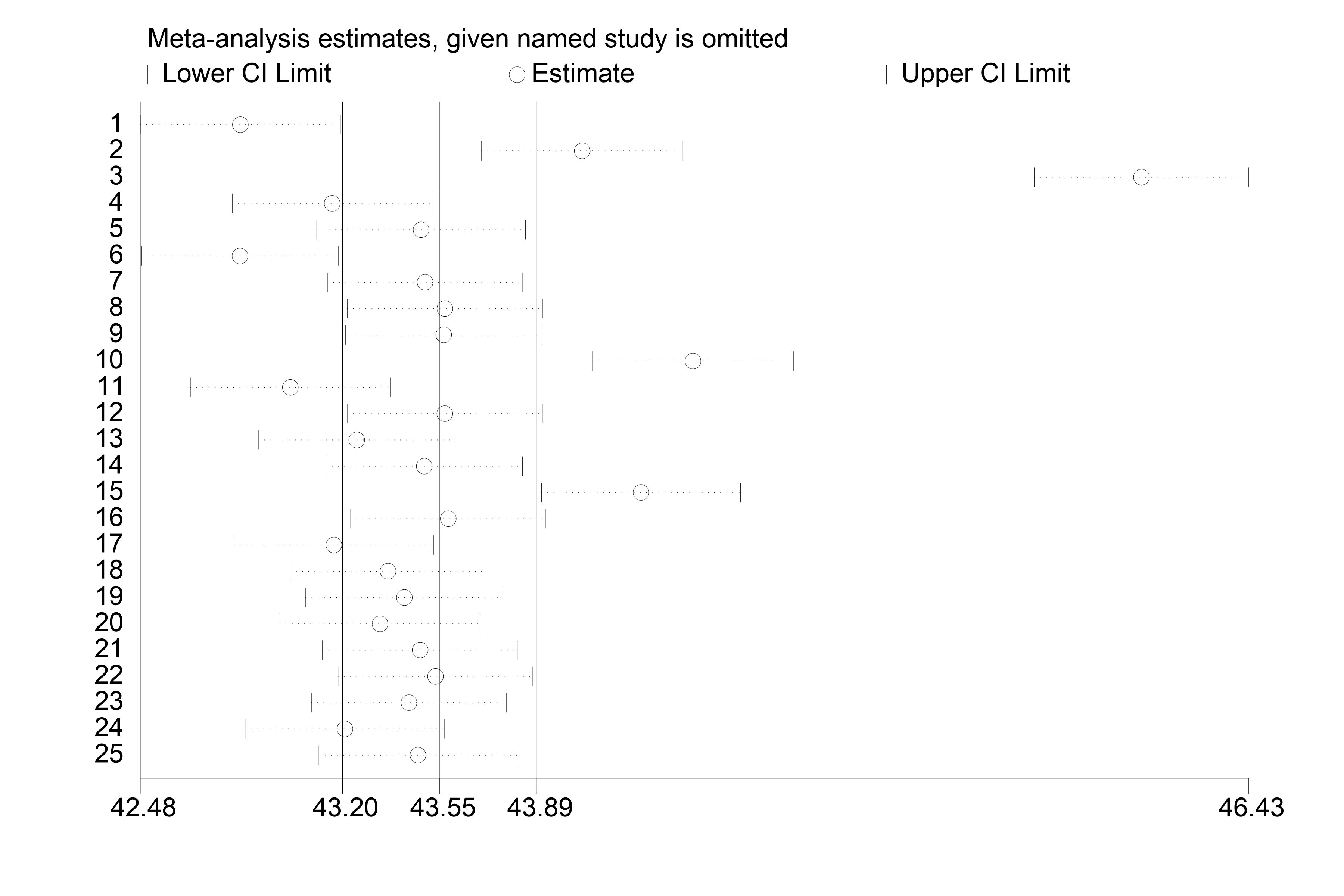

Supplement: S3 Fig — (TIFF) [file pone.0326519.s006.tiff]
